# A deep learning integrated radiomics model for identification of coronavirus disease 2019 using computed tomography

**DOI:** 10.1038/s41598-021-83237-6

**Published:** 2021-02-16

**Authors:** Xiaoguo Zhang, Dawei Wang, Jiang Shao, Song Tian, Weixiong Tan, Yan Ma, Qingnan Xu, Xiaoman Ma, Dasheng Li, Jun Chai, Dingjun Wang, Wenwen Liu, Lingbo Lin, Jiangfen Wu, Chen Xia, Zhongfa Zhang

**Affiliations:** 1 Department of Respiratory Medicine, Jinan Infectious Disease Hospital, Shandong University, 22029# Jing-Shi Road, Jinan, 250021 Shandong People’s Republic of China; 2 Institute of Advanced Research, Infervision Medical Technology Co., Ltd., 18F, Building E. Yuanyang International Center, Chaoyang District, Beijing, 100025 People’s Republic of China; 3 Department of Radiology, Jinan Infectious Disease Hospital, Shandong University, 22029# Jing-Shi Road, Jinan, 250021 People’s Republic of China; 4 Department of Radiology, Beijing Haidian Section of Peking University Third Hospital (Beijing Haidian Hospital), 29# Zhongguancun Road, Haidian District, Bejing, 100080 People’s Republic of China; 5 Department of Radiology, Inner Mongolia Autonomous Region People’s Hospital, 20# Zhaowuda Road, Hohhot, 010017 People’s Republic of China; 6 Department of Radiology, Affiliated Jinhua Hospital, Zhejiang University School of Medicine, 365# Renmin East Road, Wucheng District, Jinhua, 321000 People’s Republic of China

**Keywords:** Software, Viral infection

## Abstract

Since its first outbreak, Coronavirus Disease 2019 (COVID-19) has been rapidly spreading worldwide and caused a global pandemic. Rapid and early detection is essential to contain COVID-19. Here, we first developed a deep learning (DL) integrated radiomics model for end-to-end identification of COVID-19 using CT scans and then validated its clinical feasibility. We retrospectively collected CT images of 386 patients (129 with COVID-19 and 257 with other community-acquired pneumonia) from three medical centers to train and externally validate the developed models. A pre-trained DL algorithm was utilized to automatically segment infected lesions (ROIs) on CT images which were used for feature extraction. Five feature selection methods and four machine learning algorithms were utilized to develop radiomics models. Trained with features selected by L1 regularized logistic regression, classifier multi-layer perceptron (MLP) demonstrated the optimal performance with AUC of 0.922 (95% CI 0.856–0.988) and 0.959 (95% CI 0.910–1.000), the same sensitivity of 0.879, and specificity of 0.900 and 0.887 on internal and external testing datasets, which was equivalent to the senior radiologist in a reader study. Additionally, diagnostic time of DL-MLP was more efficient than radiologists (38 s vs 5.15 min). With an adequate performance for identifying COVID-19, DL-MLP may help in screening of suspected cases.

## Introduction

Since its first outbreak in Wuhan, China, Coronavirus Disease 2019 (COVID-19) has been extensively spreading all over the world and caused a global pandemic. Real-time reverse-transcriptase-polymerase chain reaction (rRT-PCR) amplification of SARS-CoV-2 serves as the gold standard for COVID-19 diagnosis. However, false-negative results and long turnaround time limit the clinical efficacy of rRT-PCR testing in rapid COVID-19 screening^1,2^, especially during disease outbreaks. Given that about 97% of COVID-19 patients presented chest abnormalities^1,3^, chest CT examination has been regarded as a prompt and complementary reference to rRT-PCR testing for screening COVID-19 patients^3,4^. Yet, an increasing number of chest CT examinations would overload radiologists and subtle chest abnormalities such as ground-glass opacities could be easily missed. Thus, an efficient and reliable CT-based auxiliary tool is urgently needed to help radiologists screen COVID-19 patients.

Over the past few years, different deep learning (DL)-based artificial intelligence (AI) diagnostic systems were developed and deployed in clinical practice to assist radiologists, such as the DL-based pulmonary nodules diagnostic system^5^. Since the outbreak of COVID-19, multiple machine learning (ML) and DL models for detecting lesions, assessing disease severity, and predicting disease prognosis of COVID-19 have been developed^6–13^. Wang et al. developed a DL model to provide clinical diagnosis before the pathogenic examinations by extracting radiographical features of COVID-19^8^. Yue et al. built a ML model using CT images to estimate the hospital stay of COVID-19 patients^14^. Another study developed a radiomics nomogram using features extracted from the lung parenchyma window to predict COVID-19^13^. When reviewing published literature on prediction models for COVID-19 diagnosis^15^, we noticed that regions of interest (ROIs) annotation which was time-consuming but indispensable for model development were one of the common challenges for both deep learning and radiomics modeling. Moreover, though radiomics is a widely utilized method in the field of medical imaging^16^, lack of automatic ROI annotation is a key hurdle during its clinical application because each case needs to be annotated before being applied to the radiomics models.

In recent years, radiomics is developed rapidly and has attracted broad attention for its potential to identify subtle disease characteristics that failed to be discovered by naked eyes. However, the performance of the radiomics model could be greatly influenced by different feature selection methods and classification algorithms^17–19^. To achieve the best model, feature selection and classification algorithm need to be well-designed. To our knowledge, no research so far has tried to evaluate the effects of feature selection methods and classification algorithms on the performance of radiomics models for distinguishing COVID-19 and other community acquired pneumonia (CAP) patients. In this study, we solved the time-consuming ROI annotation problem by integrating a DL segmentation algorithm with the radiomics approach, and developed an end-to-end model using CT images to screen COVID-19 patients. Additionally, cross-combinations of five feature selection methods and four machine learning algorithms were used to develop the optimal radiomics model. Furthermore, the clinical feasibility of the model was validated on an external dataset in terms of classification performance and time efficiency.

## Materials and methods

### Patients

This study was approved by the Institutional Reviewing Board (IRB) of Jinan Infectious Disease Hospital, Beijing Haidian Hospital, and Inner Mongolia Autonomous Region People's Hospital. Informed consent was waived by IRBs since patient information was anonymized to ensure privacy. All methods were carried out in accordance with relevant guidelines and regulations. For model development, a total of 293 patients (371 CT scans, some patients underwent several CT examinations) were retrospectively collected from Jinan Infectious Disease Hospital and Beijing Haidian Hospital between Jan 25 and Feb 15, 2020, including 98 COVID-19 patients, 157 other CAP patients, and 38 etiologically confirmed influenza and mycoplasma pneumonia patients. To further validate model robustness, 93 patients (31 COVID-19 patients and 62 CAP patients, 95 CT scans) were enrolled from Inner Mongolia Autonomous Region People's Hospital between Jan 26 and Feb 17, 2020, and constituted an independent external testing dataset. Of note, rRT-PCR testing for SARS-COV-2 served as the gold standard to diagnose COVID-19 patients in this study. Detailed clinical information of the enrolled patients were summarized in Table 1.

**Table 1:** Characteristics of enrolled patients and collected CT scans for model development and validation.

Overall	Training and validation set (n = 199)		Internal test set (n = 94)		External test set (n = 93)	
	COVID-19	CAP	COVID-19	CAP	COVID-19	CAP
Patients (CT scans)	72 (137)	127 (131)	26 (33)	68 (70)	31 (33)	62 (62)
Age (range)	42.0 ± 13.6 (3–78)	38.1 ± 14.4 (3–80)	42.7 ± 14.0 (15–72)	40.1 ± 19.4 (5–94)	47.5 ± 18.7 (17–85)	42.1 ± 27.4 (2–93)
Male	32 (68)	72 (75)	14 (18)	42 (44)	16 (16)	42 (42)
Female	40 (69)	55 (56)	12 (15)	26 (26)	15 (17)	20 (20)
**Jinan Infectious Disease Hospital (n = 152, scan# = 228)**						
Patients (CT scans)	41 (106)	75 (78)	19 (26)	17 (18)	–	–
Age	39.8 ± 13.6 (3–72)	35.6 ± 11.6 (20–79)	40.7 ± 11.8 (26–72)	33.1 ± 13.6 (5–65)	–	–
Male	22 (55)	48 (51)	10 (14)	12 (13)	–	–
Female	19 (51)	27 (27)	9 (12)	5 (5)	–	–
**Beijing Haidian Hospital (n = 141, scan# = 143)**						
Patients (CT scans)	31 (31)	52 (53)	7 (7)	51 (52)^a^	–	–
Age	44.8 ± 13.3 (17–78)	41.7 ± 17.1 (3–80)	48.1 ± 18.7 (15–67)	42.4 ± 20.6 (14–94)	–	–
Male	13 (13)	24 (24)	4 (4)	30 (31)	–	–
Female	18 (18)	28 (29)	3 (3)	21 (21)	–	–
**Inner Mongolia Autonomous Region People's Hospital (n = 93, scan# = 95)**						
Patients (CT scans)	–	–	–	–	31 (33)	62 (62)
Age (years)	–	–	–	–	47.5 ± 18.7 (17–85)	42.1 ± 27.4 (2–93)
Male	–	–	–	–	16 (16)	42 (42)
Female	–	–	–	–	15 (17)	20 (20)

^a^Include previously collected pathologically confirmed influenza pneumonia (20 cases) and mycoplasma pneumonia (18 cases) which serves as independent test data.

In addition, patients’ characteristics were summarized, including clinical stages and imaging manifestations. In particular, over 65% of the included COVID-19 patients were clinically classified as the moderate type, followed by 27.1% mild type, 2.3% severe type, and 0.8% critical type (Appendix Table S1). In terms of imaging manifestations on chest CT scans, multifocal small patchy shadows, ground glass opacity (GGO), and consolidation were the main lesions found in both COVID-19 and CAP cases. As can be seen in Appendix Table S2, GGO was more common and consolidation was less common in COVID-19 patients than among CAP cases, which could be attributed to the relatively larger proportion of mild or moderate clinical type patient. Other reported imaging manifestations, including infiltrate and pleural effusion, were rare among the included patients of this study.

### DL segmentation algorithms

**Figure 1:**
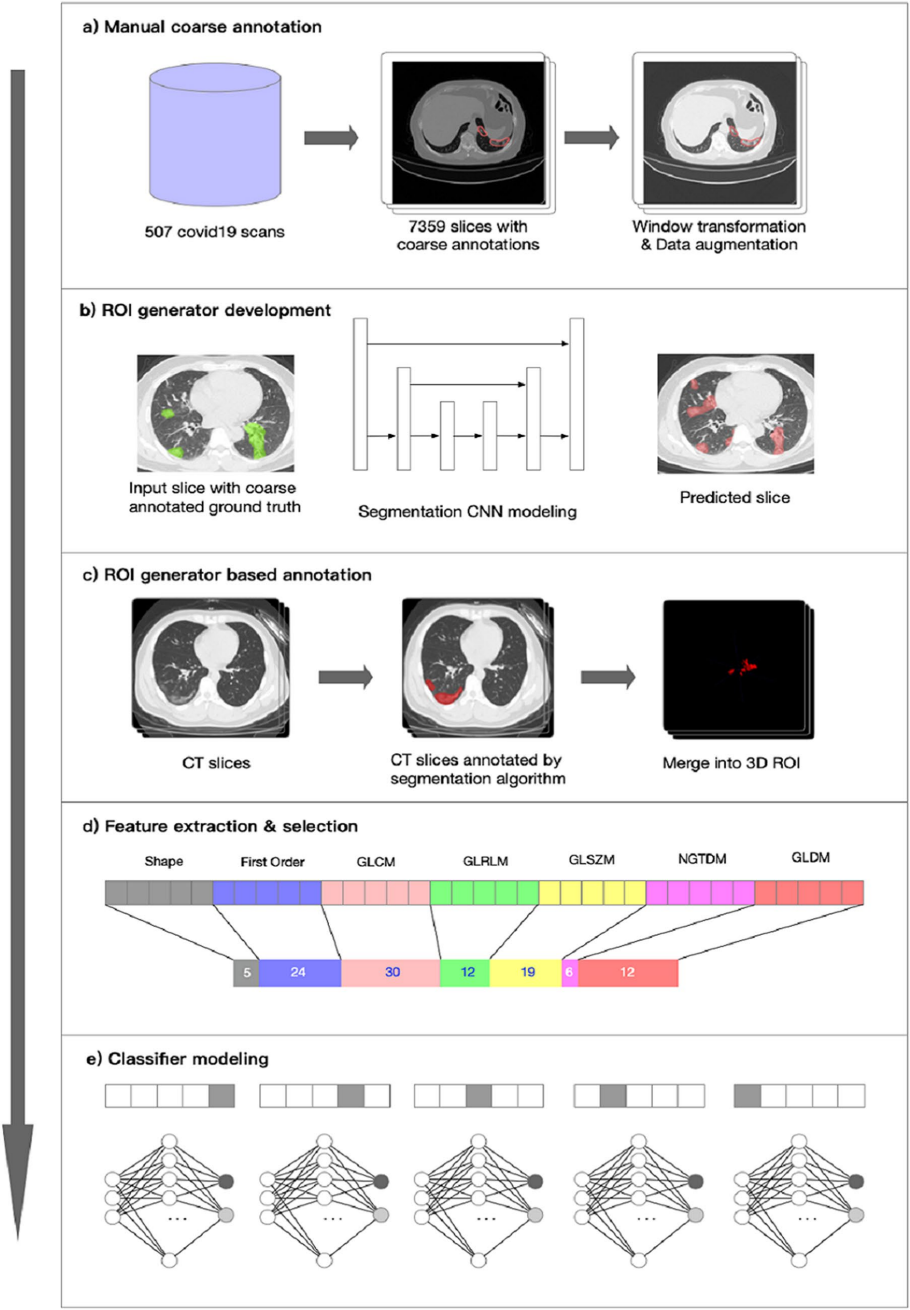
The workflow of the end-to-end model development. Manual coarse annotation was performed on 507 CT scans which were further utilized to develop the deep learning (DL) segmentation algorithm (**a**,**b**). The segmentation algorithm was applied to annotate lesions on CT scans in this study; annotations on each CT slices were eventually merged into a 3D ROI (**c**). Feature extraction and selection were performed using pyRadiomics and different selection methods; the optimal method L1-LR selected 108 features of different categories for radiomics modeling (**d**). Five-fold cross-validation was utilized for modeling (**e**).

The DL segmentation algorithm was a built-in feature on InferScholar platform by Infervision (https://www.infervision.com/, Beijing, CHINA) and applied to automatically delineate ROIs in this study. The segmentation algorithm was trained with 507 sets of CT scans from suspected COVID-19 patients in Wuhan area. Coarse annotation strategy was utilized in which major lesions with multifocal small patchy shadowing, ground-glass opacities, and consolidations were selectively annotated on CT images by experienced radiologists (Fig. 1a). During algorithm training, CT images of different sizes were first resized to 512 × 512 using bilinear interpolation method as previously described^20^ and the CT values of images were rescaled at window center of -600 and window width at 1500 so that the pneumonia lesions could be presented and easily distinguished (Fig. 1b). Annotated lesions on each slide were merged into a 3D ROI after segmentation (Fig. 1c). Training and testing of the DL segmentation algorithm were performed by using Mxnet (version 1.6.0) and CUDA (version 10.0).

To briefly summarize the structure of the DL segmentation algorithm, U-Net was the main architecture of the algorithm in which Xception^21,22^ served as the backbone (sFig. 1). The annotation performance was evaluated by the Dice index. Dice Loss equation for loss function was as followed:

$$Dice Loss= 1-\frac{2*Pred*Anno}{Pred+Anno}$$

where *Pred* denotes lesion pixels predicted by the DL segmentation algorithm and *Anno* represents the reference lesion pixels annotated by senior radiologists.

### Feature extraction

In this study, we used Python (version 3.8.1) to call the pyRadiomics package (version 2.2.0) for radiomics feature extraction. A total of 1454 features were extracted from the DL algorithm segmented ROIs and can be subdivided into 7 classes, including first-order (FOS), shape, Gray Level Cooccurence Matrix (GLCM), Gray Level Run Length Matrix (GLRLM), Gray Level Size Zone Matrix (GLSZM), Neighbouring Gray Tone Difference Matrix (NGTDM), and Gray Level Dependence Matrix (GLDM) features. Detailed information on feature extraction methods and parameters^23^, and the number of extracted features for each feature class was summarized in Appendix Table S3.

### Feature selection

In order to select discriminating features, five methods were applied and compared in this study, including L1 regularized least absolute shrinkage and selection operator (L1-LASSO), L1 regularized logistic regression (L1-LR), L1 regularized ridge regression (L2-Ridge), eXtreme gradient boosting (XGBoost), and Z-test^24,25^. Five-fold cross-validation method was utilized. All methods were implemented by calling the scikit-learn (version 0.20.2) package and the optimal one with the highest accuracy was chosen as the final dimensionality reduction method.

### ML model training and testing

For unbiased estimation of diagnostic accuracy, data from two hospitals (Jinan Infectious Disease Hospital and Beijing Haidian Hospital) was divided into training and internal testing sets at a ratio of 2:1; data from the third hospital was utilized as an external testing set. With the selected features, four independent ML models were trained on the training set, including support vector machine (SVM), multi-layer perceptron (MLP), logistic regression (LR), and XGBoost. These methods were all implemented by calling the scikit-learn (version 0.20.2) package. To select the best model and the optimal hyper-parameters for each model, five-fold cross-validation was performed on the training set, in which 80% of the data was randomly selected to train models and the remaining 20% data (tuning set) validated the trained models. Training and validation process repeated five times until each cross section was part of the tuning set once. In model testing stage, ensemble models from five-fold-cross validation were used to discriminated COVID-19 and CAP patients while the model performance was evaluated on internal and external testing datasets.

### Reader study

To further evaluate the clinical feasibility of these proposed models, two radiologists (one senior radiologist with 15 years’ experience and one junior radiologist with 5 years’ experience) participated in the reader study on both the internal and external testing datasets. The senior radiologist and junior radiologist both had taken part in the fight against COVID-19 in the front line. They diagnosed cases independently only based on the CT imaging information in the reader study. Their diagnostic performance was compared with the proposed end-to-end models. Of note, the diagnostic efficiency was evaluated in terms of diagnostic time-consumption.

### Model evaluation and statistical analysis

Diagnostic performance was evaluated by classification sensitivity, specificity, precision, accuracy, F1 score, G-Mean, and area under ROC curve (AUC) and PR curve (AP). PR curve, a measure complementary to the ROC curve^26^, was utilized as well just in case of the possible asymmetrical data problems. Categorical variables were expressed in terms of frequency and statistically analyzed by Chi-square test. P < 0.05 was considered statistically significant. Continuous variables were represented by the means ± SD. A two-sided 95% confidence interval for AUC or AP was constructed following the approach of Hanley and McNeil (1982)^27^. Cohen’s Kappa coefficient was calculated to measure the agreement between ground-truth results and model predictions. All statistical analyses were performed with the R statistical package (The R Foundation for Statistical Computing, Vienna, Austria).

## Results

### Performance of feature selection methods and ML models

**Figure 2:**
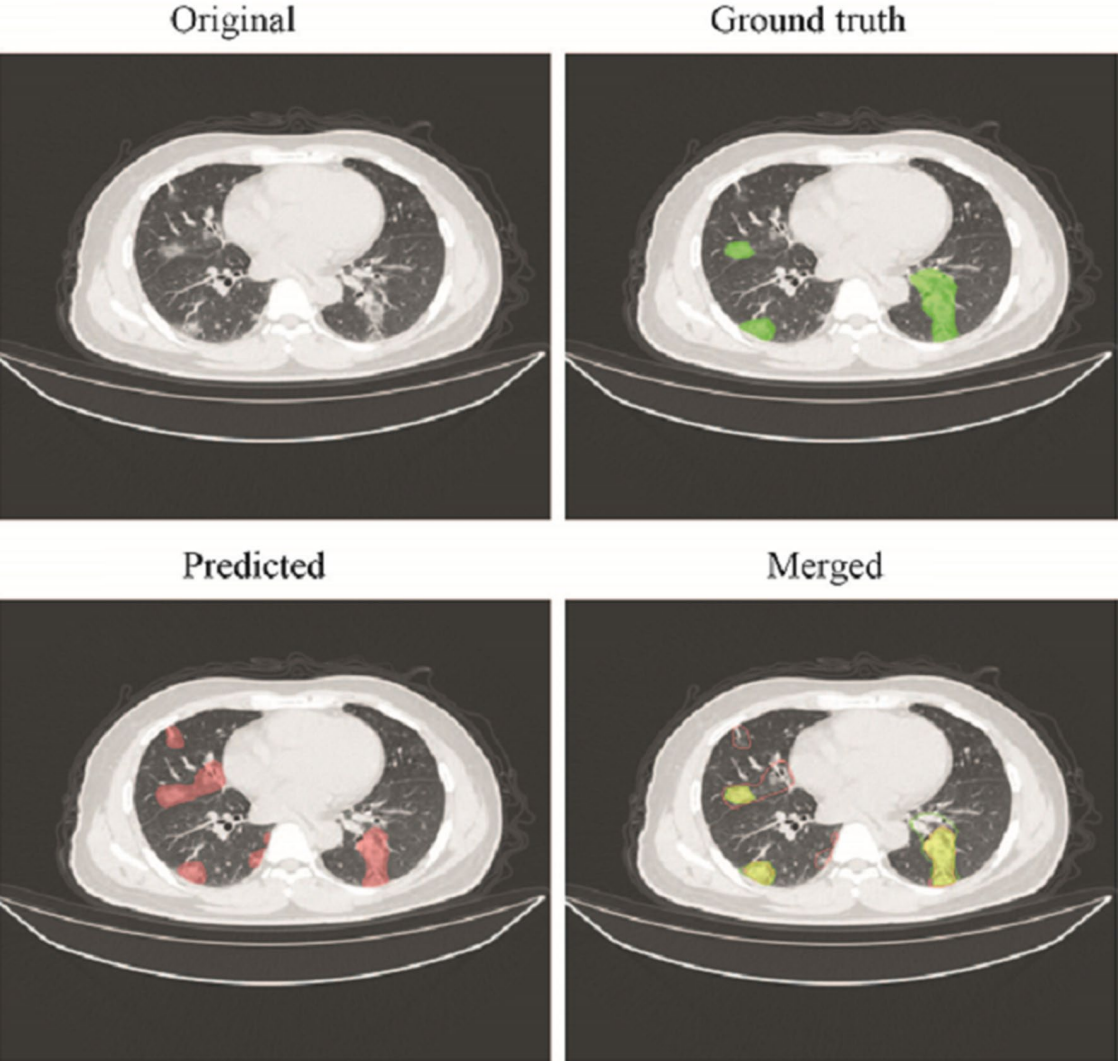
The representative of coarse annotated (Green) and AI labeled (Red) ROIs. Although trained with coarse annotated slices, DL-based segmentation algorithm could recognize and delineate most of the lesions on CT scans in the testing datasets.

**Figure 3:**
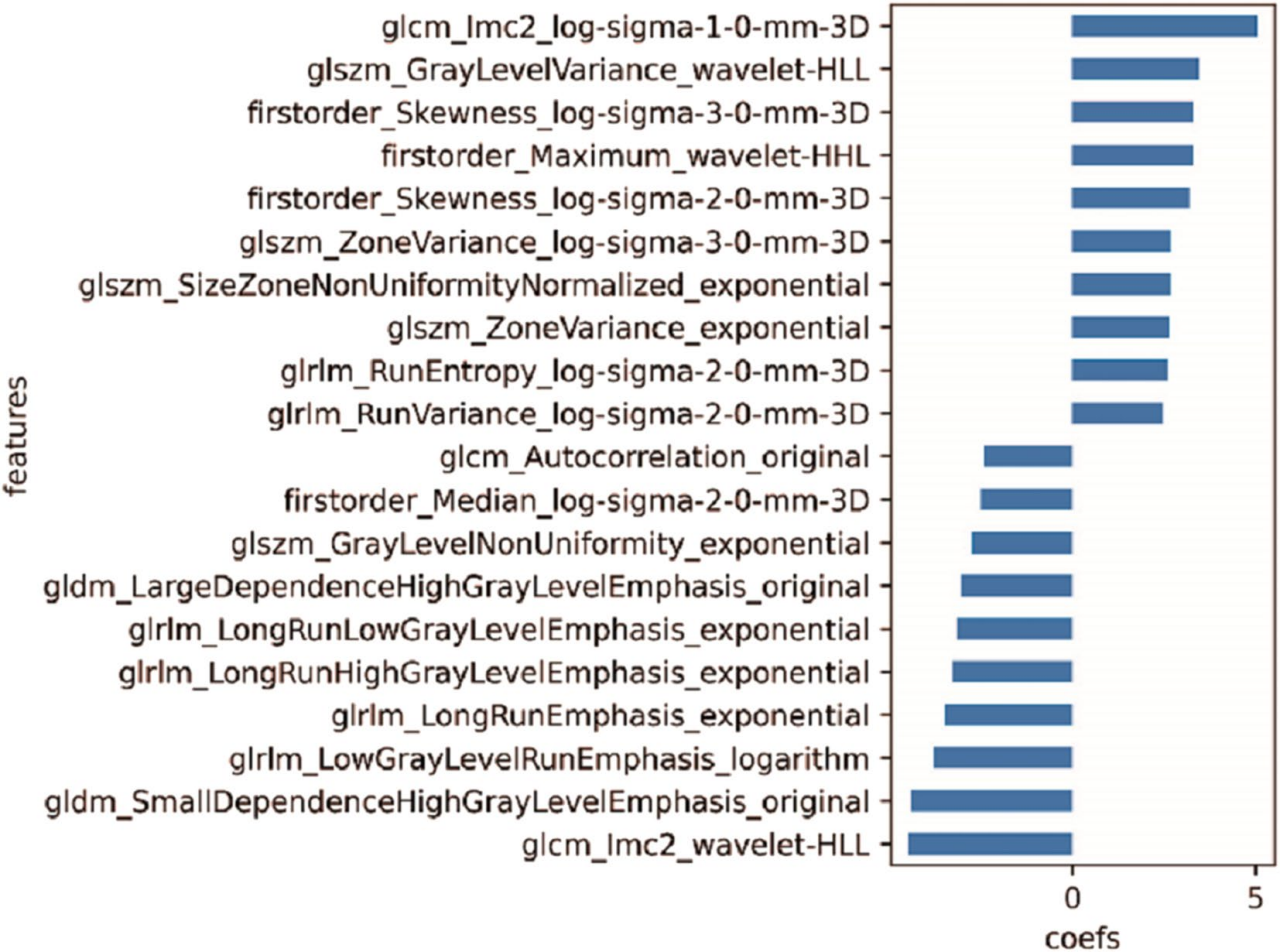
The representative of selected radiomics features. A total of 108 features were selected from 1454 extracted ones with L1-LR method. Features with the top 20 absolute coefficients was shown in this figure, while details for entire selected features were listed in Appendix Table S6.

The pre-trained DL segmentation algorithm achieved a Dice index of 0.69 and also displayed an adequate performance on the CT scans in this study. Much more lesions were annotated by DL algorithms comparing the coarse annotation method. Examples of coarse annotated and AI labeled ROIs were shown in Fig. 2. Of the five selection methods, L1-LR which selected 108 radiomics features enabled three ML models to achieve the highest AUC on validation set and was thus selected as the optimal method (sFig. 2, Fig. 1d). Pearson Correlation Coefficient (PCC) among the 108 selected features were calculated; features with PPC < 0.8 and 0.5 constituted another two feature sets, respectively (Appendix Tables S4 and S5). Feature redundancy was examined by training models with these three features sets and it turned out that 108 features guaranteed the optimal model performance (sFig. S, Figs. 5a, and 6a). All selected features were listed in Appendix Table S6 while features with the top 20 absolute coefficients were shown in Fig. 3 as the representatives.

**Figure 4:**
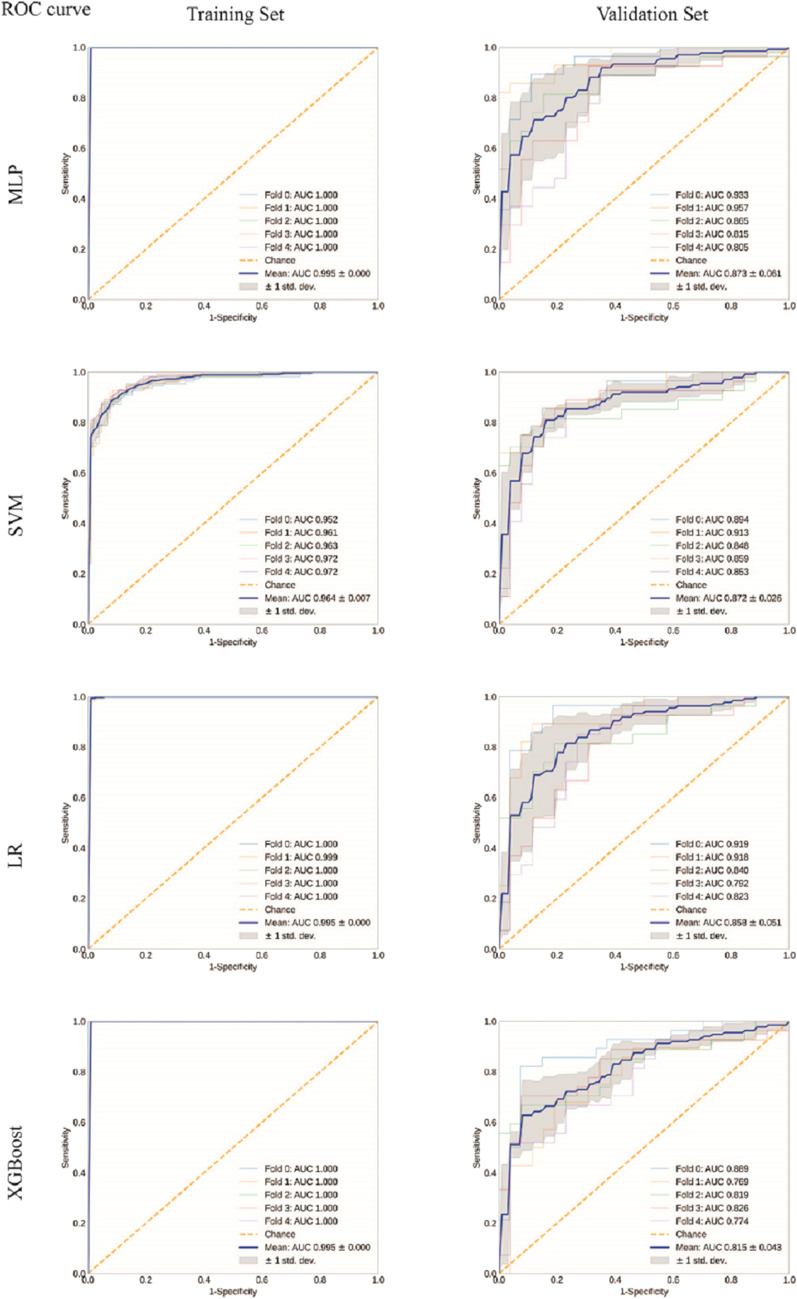
Receiver operating characteristic (ROC) curve analyses of ML models on training and validation sets. ROC curves were analyzed on training and validation sets to evaluate the performance of ML models. Except SVM, all the other models presented perfect fitting on training set while MLP displayed the optimal performance on the validation set.

After training, MLP, SVM, LR, and XGBoost obtained a mean AUC of 0.995, 0.964, 0.995, and 0.995 on the training set; the higher the AUC on training set, the better the model fit. Meanwhile, the mean AUC of 0.873 (95% confidence interval (CI) 0.812–0.934), 0.872 (95% CI 0.846–0.898), 0.858 (95% CI 0.807–0.909), and 0.815 (95% CI 0.772–0.858) were obtained on validation set, respectively (Fig. 4, sFig. 4). L1-LR + classifier MLP (DL-MLP) demonstrated the optimal performance during the training.

### Performance evaluation of the end-to-end models

**Figure 5:**
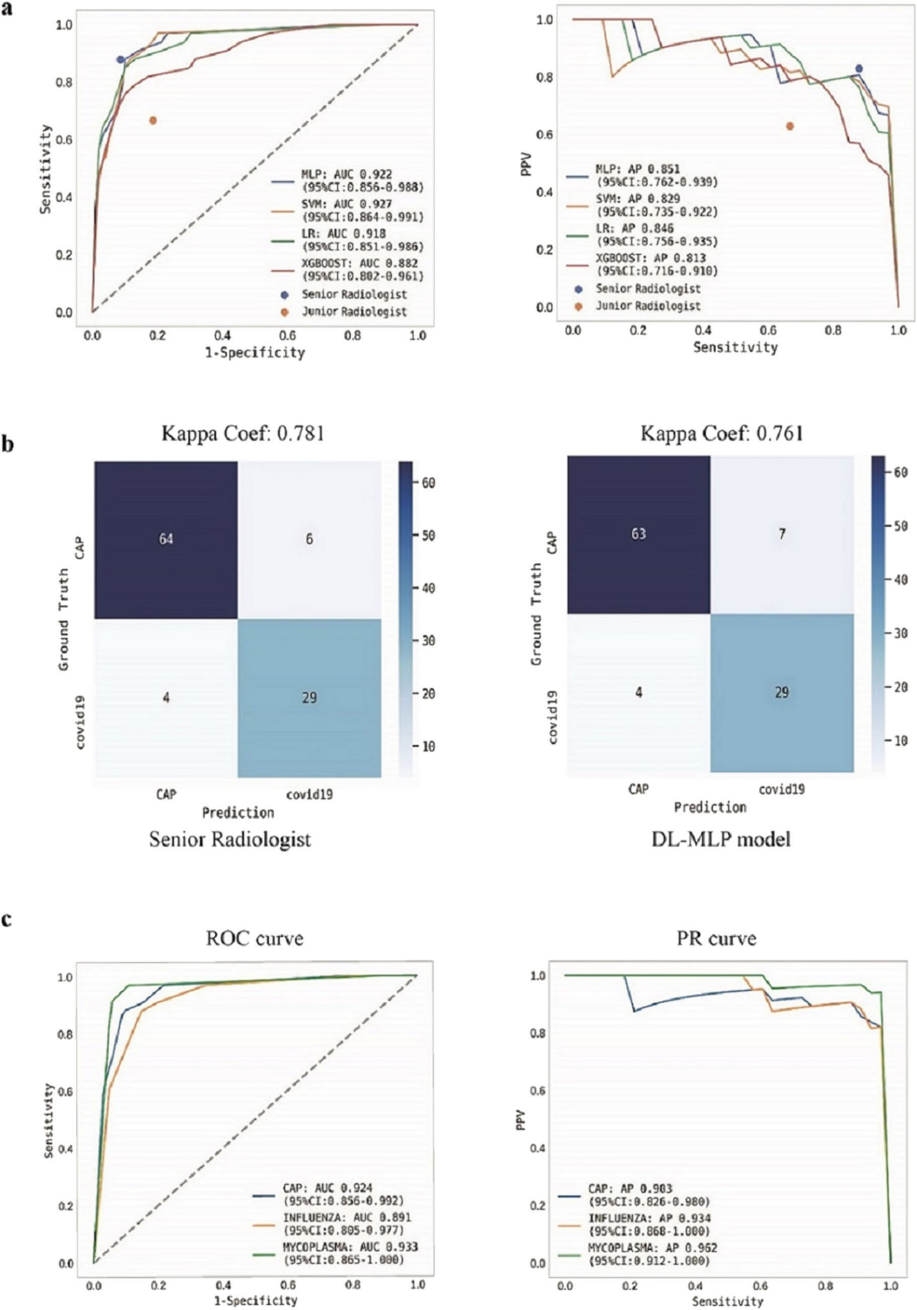
Performance of ML models and radiologists on the internal testing dataset. (**a**) ROC and precision-recall (PR) curve analyses were performed for DL-ML models. The performance of radiologists was dotted according to their sensitivity and specificity. (**b**) Confusion matrices for binary classification of COVID-19 and other community-acquired pneumonia (CAP). The exact number of true positives, false positives, true negatives, and false negatives were listed. (**c**) ROC and PR curve analyses on independent internal test data. A batch of etiologically confirmed influenza and mycoplasma pneumonia data was utilized in the internal testing dataset. DL-MLP displayed an adequate performance in distinguishing COVID-19 from them.

ML models integrated with DL segmentation algorithm constituted the end-to-end models. We then evaluated the performance of these models on testing datasets. DL-MLP outperformed other models with an AUC of 0.922 (95% CI 0.856–0.988), an F1 score of 0.841, and a kappa coefficient of 0.761 on the internal testing dataset; the AP reached 0.851 (95% CI 0.762–0.939) (Fig. 5a,b). In contrast, the AUC of DL-SVM, DL-LR, and DL-XGBoost were 0.927 (95% CI 0.864–0.991), 0.918 (95% CI 0.851–0.986), and 0.882 (95% CI 0.802–0.961), respectively. Detailed diagnostic performance metrics of these models were listed in Table 2. In addition, subgroup analysis was performed between COVID-19 and etiologically confirmed influenza pneumonia or mycoplasma pneumonia and DL-MLP again demonstrated an adequate classification performance with AUC of 0.891 (95% CI 0.805–0.977) and 0.933 (95% CI 0.865–1.000) (Fig. 5c).

**Table 2:** Detailed diagnostic metrics of end-to-end models and radiologists on internal and external testing datasets.

	Accuracy	Sensitivity	Specificity	Precision	F1-score	G-mean
**Internal testing dataset**						
MLP	0.893^a^	0.879^a^	0.900	0.806	0.841	0.889
SVM	0.845^a,b^	0.909^a^	0.814	0.698	0.789	0.860
LR	0.874^a^	0.848^a,b^	0.886	0.778	0.812	0.867
XGBoost	0.816^a,b^	0.788 ^a,b^	0.829	0.684	0.732	0.808
Senior R	0.903^a^	0.879^a^	0.914	0.829	0.853	0.896
Junior R	0.767^b^	0.667^b^	0.814	0.629	0.647	0.737
P value	< 0.05*	< 0.05*	> 0.05	> 0.05	–	–
**External testimg dataset**						
MLP	0.884^a,b^	0.879^a,b^	0.887^a^	0.806^a,b,c^	0.841	0.883
SVM	0.758^c^	0.970^b^	0.645^b^	0.593^d^	0.736	0.791
LR	0.905^a,b^	0.970^b^	0.871^a^	0.800^c^	0.877	0.919
XGBoost	0.495^d^	0.758^a^	0.355^c^	0.385^e^	0.510	0.518
Senior R	0.926^b^	0.818^a^	0.984^d^	0.964^b^	0.885	0.897
Junior R	0.832^a,c^	0.818^a^	0.839^a^	0.730^a,c,d^	0.771	0.828
P value	< 0.05*	< 0.05*	< 0.05*	< 0.05*	–	–

*On either internal or external testing dataset, different lowercase letters in the same column indicate significant differences among different models or readers (P < 0.05).

**Figure 6:**
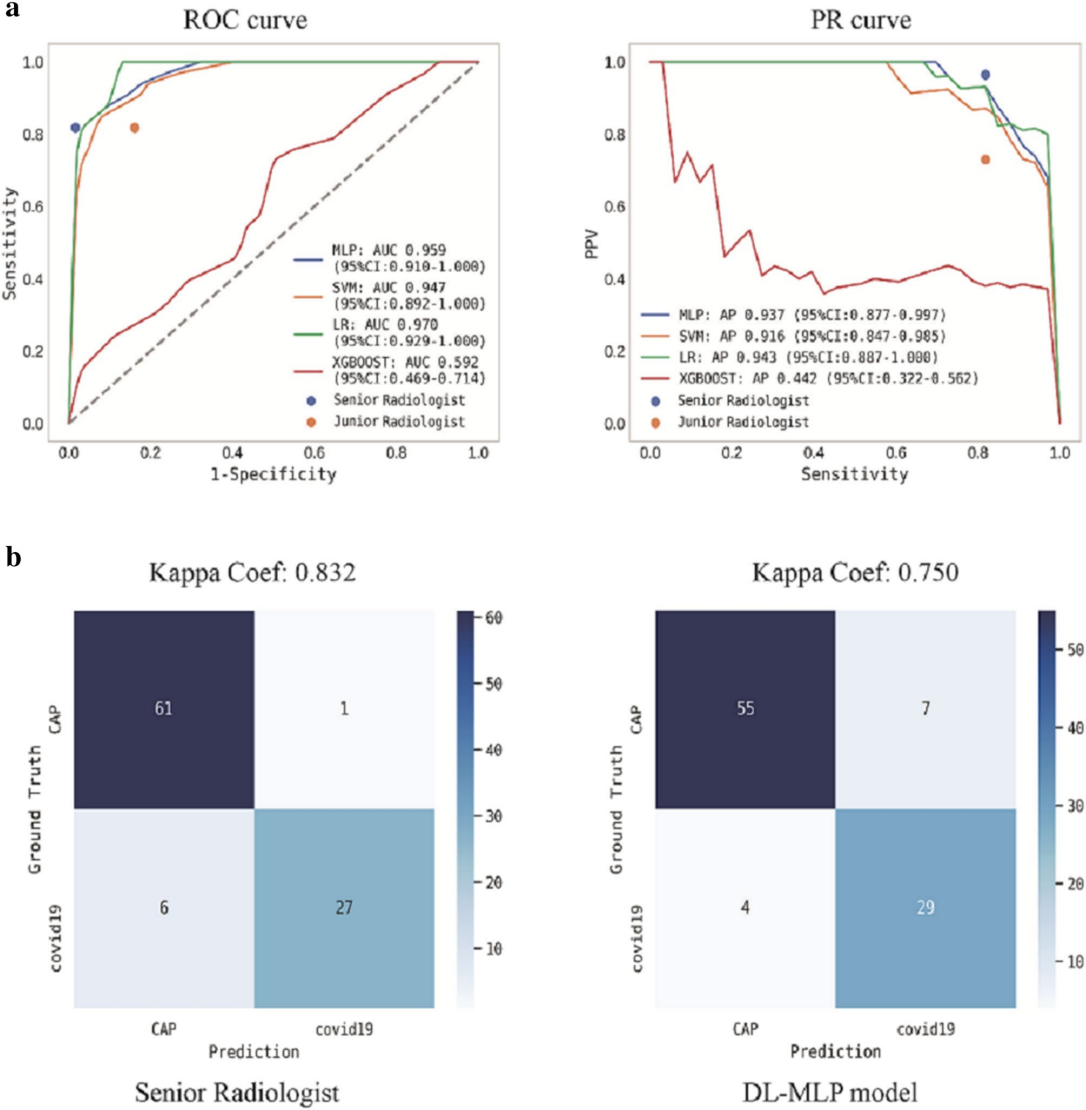
Performance of ML models and radiologists on the external testing dataset. (**a**) ROC and PR curves analyses were performed. Ensemble curves of ML models were plotted while the performance of radiologists was dotted according to the sensitivity and specificity. (**b**) Confusion matrices for binary classification of COVID-19 and CAP. The exact number of true positives, false positives, true negatives, and false negatives were listed.

Furthermore, DL-MLP achieved better performance on the external testing dataset with an AUC of 0.959 (95% CI 0.910–1.000), an F1 score of 0.841, and a kappa coefficient of 0.750; its AP reached 0.937 (95% CI 0.877–0.997). Detailed diagnostic performance metrics of other models were summarized in Table 2 and Fig. 6. Notably, it just took the end-to-end model 38 s to diagnose each input CT scan, indicating its high efficiency in practice.

### Performance evaluation of the participated radiologists in a reader study

In comparison to the junior radiologist, senior radiologist achieved an overall better performance with the diagnostic accuracy, precision, sensitivity, and specificity of 0.90, 0.83, 0.88, and 0.91 on the internal testing dataset and 0.926, 0.964, 0.818, and 0.984 on external testing dataset (Table 2). The radiologists’ diagnostic performance was dotted in ROC and PR curves according to their sensitivity, specificity, and precision (Figs. 5a and 6a). The kappa coefficient of senior radiologist reached 0.781 and 0.832 on internal and external testing datasets (Figs. 5b and 6b). In addition, junior and senior radiologists spent an average time of 5.29 min and 5 min to diagnose a set of CT images.

## Discussion

Early and timely detection of COVID-19 patients is of great importance in containing the pandemic. The practice has proved that the CT examination serves as a complementary approach to rRT-PCR for COVID-19 screening in some emergent scenarios^28–30^. By integrating DL segmentation algorithm with radiomics, we developed an end-to-end model using CT images from multiple medical centers to screen COVID-19 patients. Automatically delineated ROIs by DL segmentation algorithm greatly enhanced the application potentials of radiomics models in clinical practice. Trained with selected radiomics features, DL-MLP model demonstrated comparable diagnostic performance to a senior radiologist with 15 years’ experience on internal and external testing datasets.

To date, many DL and radiomics models were developed since the outbreak of COVID-19, focusing on screening, diagnosis, and prognosis of COVID-19^15^. However, due to limited medical labor resources and diffused lesion distribution across multiple sections, ROI annotations remained challenging in many of the current studies^8,9,11^. In our study, we utilized a DL segmentation algorithm that was trained with 507 sets of coarse annotated suspected COVID-19 CT scans. Lesions were selectively annotated on certain CT sections where they predominantly presented. This strategy reduced the annotation workload when medical resources were scarce and eventually achieved adequate results. The DL segmentation algorithm enabled direct application of radiomics models in clinical practice by saving the need for manual annotation, which is of great value to be extended to other disease scenarios when the radiomics approach was utilized.

Of note, five feature selection methods and four machine learning algorithms were utilized so as to discover the optimal radimocis model for identifying COVID-19 patients. A total of 20 models were tested and compared on both internal and external testing datasets in terms of AUC. Optimal feature selection methods were firstly screened by comparing the corresponding model performance on validation sets. Three of the four machine learning models achieved the best AUC when trained with L1-LR selected features. Redundancy of L1-LR selected features was further tested by modeling without features with strong correlations (PCC ≥ 0.8; PCC ≥ 0.5). All L1-LR selected features were finally utilized because of the robust performance on internal and external testing datasets. Machine learning models were trained with L1-LR selected features. Based on the performance on internal and external testing datasets in terms of AUC, AP, and other diagnostic performance metrics, the optimal model MLP was further analyzed in subgroups and compared with radiologists.

Current diagnostic performance for COVID-19 varied from model to model due to different development datasets and techniques. Detection sensitivity ranged from 0.83 to 1 while the AUC ranged from 0.81 to 0.996^15,31,32^. A recent study ensembled transfer learning with deep convolutional neural networks (15 architectures) to detect COVID-19 on CT images and achieved the best performance with sensitivity of 0.854, accuracy of 0.85, and precision of 0.857^33^. Another DL-based multi-view fusion model was developed using CT images with the maximum lung regions in axial, coronal and sagittal views and achieved AUC, accuracy, sensitivity and specificity of 0.819, 0.760, 0.811 and 0.615 on testing set, respectively^32^. In comparison, our study shared similar data size and achieved a better diagnostic performance as evidenced by the AUC, accuracy, sensitivity and specificity of 0.959, 0.884, 0.879 and 0.887 on the external testing dataset. Similarly, the multi-view fusion model solved annotation problem by using certain whole CT images, however, that may also result in insufficient features to properly detect COVID-19^32^. Another deep learning model was trained with a large dataset to identify COVID-19 from other pneumonia^34^. Like this model, our proposed DL-MLP could also distinguish COVID-19 from etiologically confirmed influenza and mycoplasma pneumonia and achieved better performance in terms of AUC.

Notably, there were also developed radiomics models to distinguish COVID-19, predict hospital stay, disease severity, and prognosis of COVID-19 patients^10,12–14^. An earlier radiomics study that utilized both lesion and normal region patches cropped from COVID-19 CT scans achieved a higher classification accuracy of 99.68% with GLSZM features^35^. However, this study ignored the within-patient correlation between the two classes of image patches. Meanwhile, radiomics nomogram for predicting COVID-19 was also developed by combining radiomics scores and significantly associated CT characteristics^13^ and obtained a comparable performance to ours. Yet, note that in addition to internal and external testing sets, the proposed DL-MLP model was further validated by comparing with experienced radiologists on external testing dataset, which substantiated the model’s greater application potentials in clinical scenarios.

The diagnostic performance of two radiologists served as the benchmark to evaluate the diagnostic efficacy of models in this study. Unlike studies with imbalanced classifications of data whose diagnostic threshold was determined by G-Mean^36^, our model output the normalized predicted probabilities of each class and achieved an adequate performance on identifying COVID-19 with a diagnostic threshold of 0.5 (sFig. 5). Notably, diagnostic performance of the participating radiologists on identification of COVID-19 was generally comparable to radiologists in other studies with similar sensitivity, specificity and accuracy^11,37^. In consistent with previous DL studies^11,37,38^, DL-MLP demonstrated comparable diagnostic performance to the experienced senior radiologist on both internal and external testing datasets in terms of detection sensitivity, specificity and accuracy. Adequate performance on the external testing dataset further increased the reliability of the end-to-end DL-MLP model. In addition, diagnostic efficiency is another important parameter to evaluate model feasibility. Comparable reading time of the radiologists was found in the current and previous study (5.15 min vs. 6.5 min)^11,38^; in contrast, the model made a diagnosis in about 38 s which was much more efficient.

There are still limitations in this study that can be improved in future research. More radiologists for reader study, the utilization of AI-assisted reading mode, and detailed subgroup analyses could further validate the model’s feasibility in clinical practice. In addition, integrating clinical information other than CT images could potentially improve diagnostic performance.

In conclusion, an end-to-end DL-MLP model was developed by integrating the DL segmentation algorithm with the radiomoics approach to efficiently screen COVID-19 patients from other CAP patients. DL-MLP achieved an adequate diagnostic performance that was comparable to a senior radiologist on both internal and external testing datasets, demonstrating the algorithm’s great potential to assist radiologists to screen suspected COVID-19 cases in joint with rRT-PCR testing in emergent scenarios or high prevalence areas.

## Supplementary Information

Supplementary Information 1.

Supplementary Information 2.

## Data Availability

The data will be made available to others on reasonable requests to the corresponding author.
