# Supplementary material for: A deep learning integrated radiomics model for identification of coronavirus disease 2019 using computed tomography
Source: Sci Rep. 2021 Feb 16;11:3938. doi: 10.1038/s41598-021-83237-6 (PMC7886892; doi:10.1038/s41598-021-83237-6)
Supplement: Supplementary file 1 — Supplementary Information 1. [file 41598_2021_83237_MOESM1_ESM.pdf]

Supplementary figures

Title: A Deep Learning Integrated Radiomics Model for Identification of Coronavirus Disease 2019 Using Computed Tomography

Authors: Xiaoguo Zhang[1§], Dawei Wang[2§], Jiang Shao[3], Song Tian[2], Weixiong Tan[2], Yan Ma[1], Qingnan Xu[1], Xiaoman Ma[1], Dasheng Li[4], Jun Chai[5], Dingjun Wang[6], Wenwen Liu[3], Lingbo Lin[3], Jiangfen Wu[2], Chen Xia[2], Zhongfa Zhang[1*]

Manuscript tracking number: SREP-20-02072A

sFig. 1

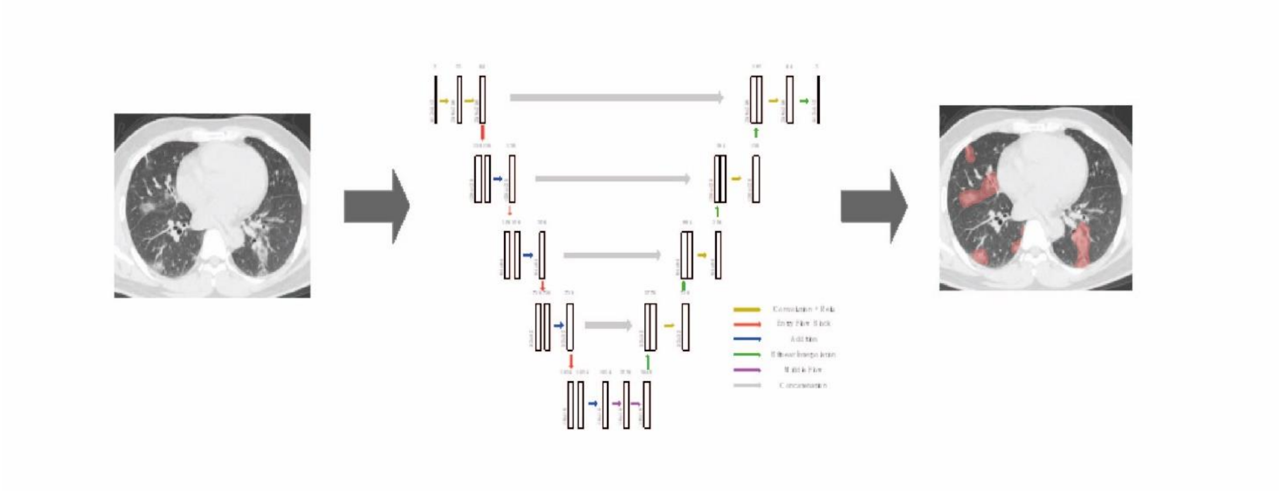

sFig. 1. Architecture of U-Net for infected lesion segmentation. A total of 507 CT scans from suspected COVID-19 patients in Wuhan area constituted the training dataset. Coarse annotation was performed by experienced radiologists, in which multifocal small patchy shadowing, ground-glass opacities, and consolidations were selectively annotated on CT images with major lesions. In subsequent, 7359 slices were utilized to train the DL segmentation algorithm.

sFig.2a

ROC curve

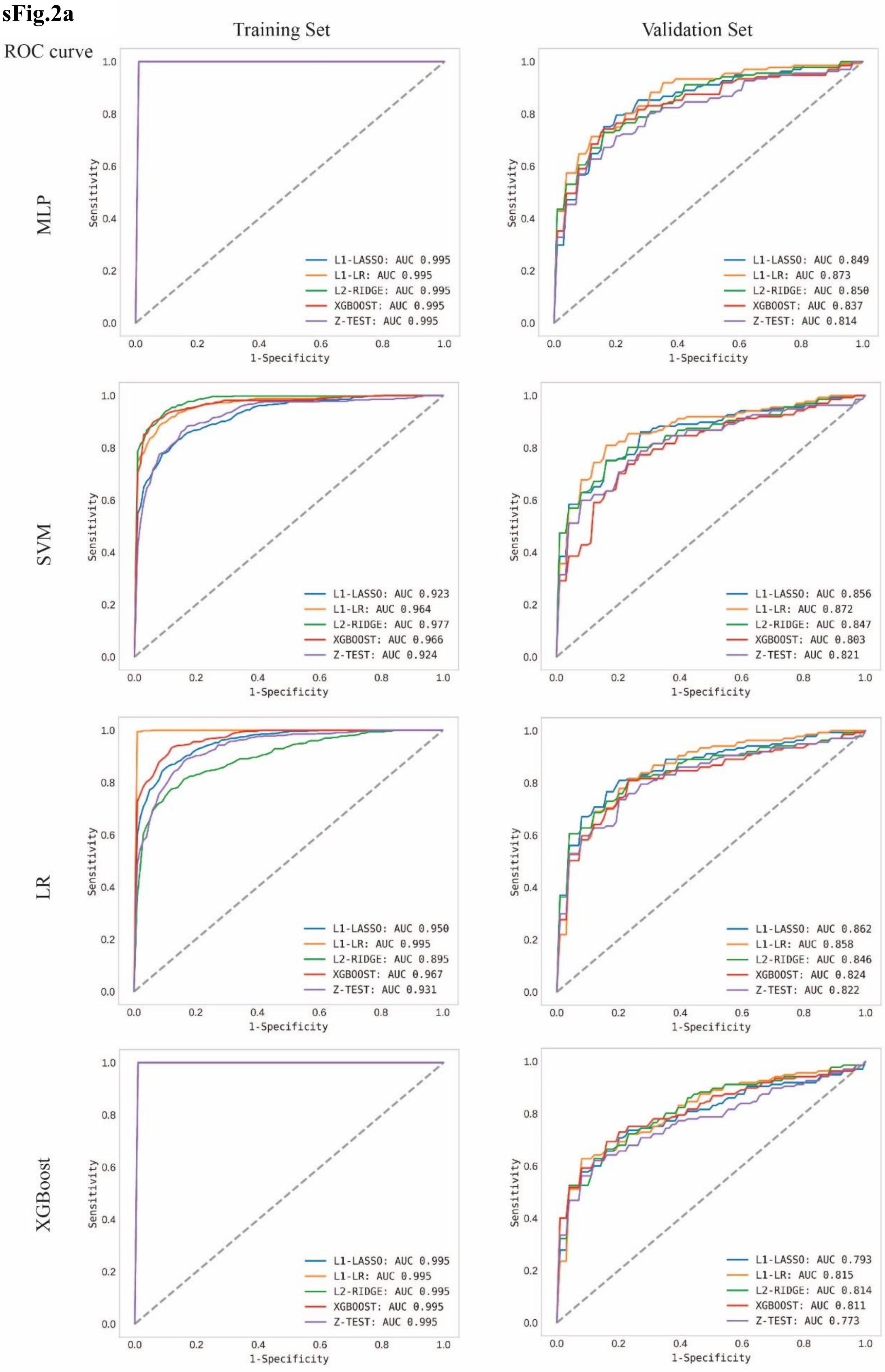

sFig.2b

PR curve

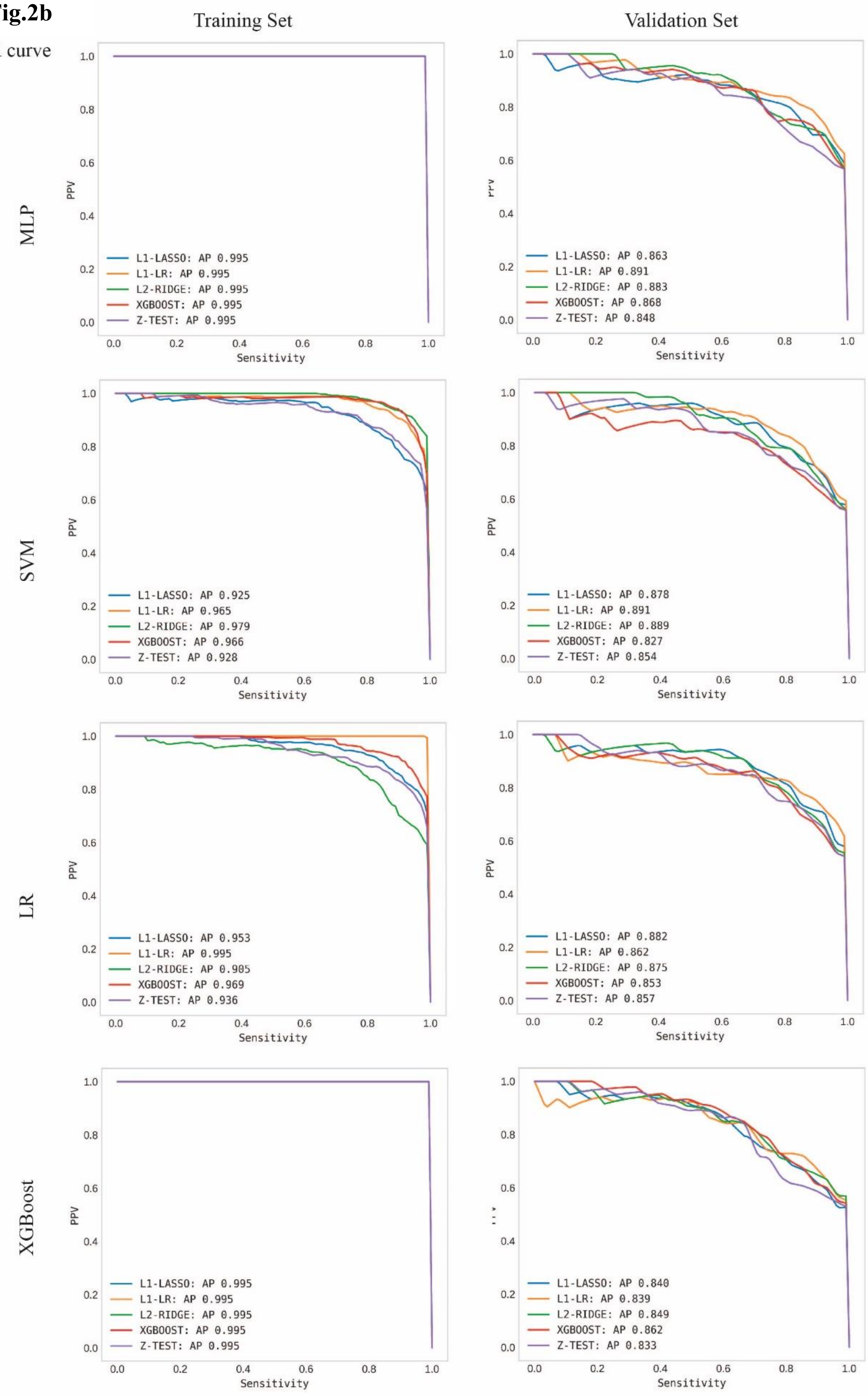

sFig.2. Effectiveness of different radiomic features selection methods. In order to find out the optimal feature selection approach for our study, five representative methods were utilized for feature selection, including L1 regularized least absolute shrinkage and selection operator (L1-LASSO), L1 regularized logistic regression (L1-LR), L1 regularized ridge regression (L2-Ridge), eXtreme gradient boosting (XGBoost), and Z-test. By analyzing the ROC curves (a) and PR curves (b) on the training and validation sets, the performance of corresponding developed models is evaluated to verify and compare the effectiveness of different feature selection methods.

sFigure 3

a

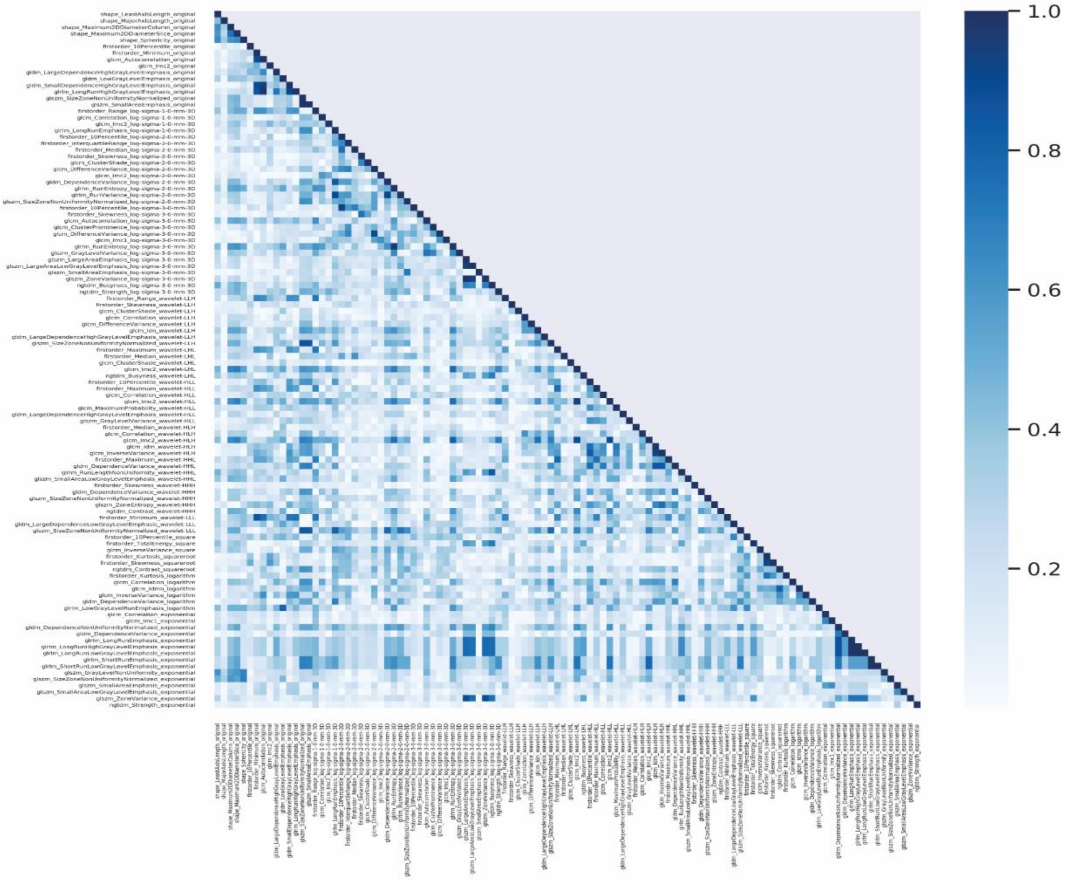
1.0
0.8
0.6
0.4
0.2

b

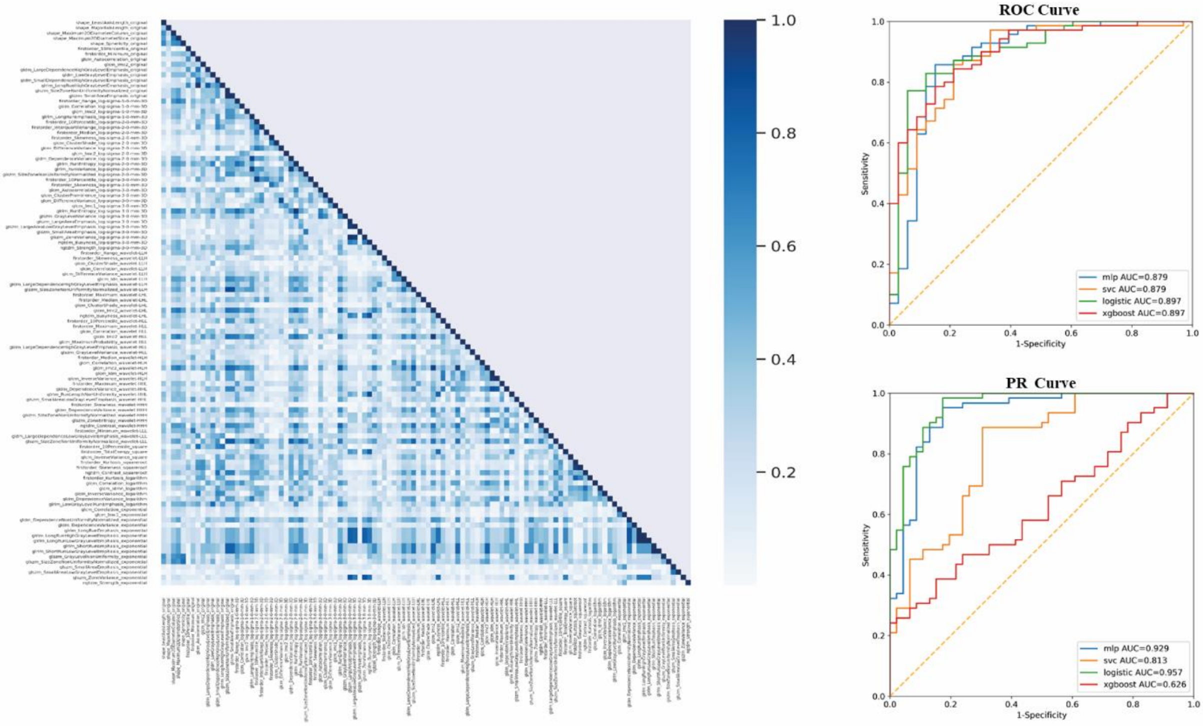
1.0
0.8
0.6
0.4
0.2
ROC Curve
Sensitivity
1-Specificity
mlp AUC=0.879
svc AUC=0.879
logistic AUC=0.897
xgboost AUC=0.897
PR Curve
Sensitivity
1-Specificity
mlp AUC=0.929
svc AUC=0.813
logistic AUC=0.957
xgboost AUC=0.626

sFigure 3

c

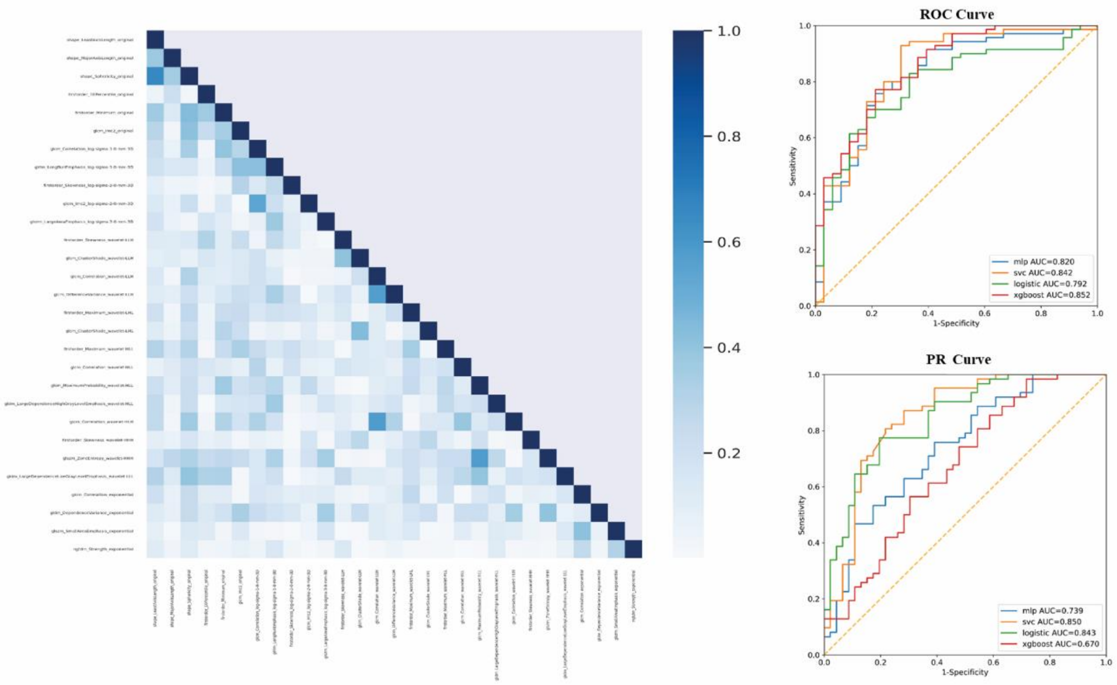

sFig.3. Redundancy analysis for selected radiomics features. Pearson Correlation Coefficient (PCC) among the 108 selected features were calculated. All features, features with PPC < 0.8 and 0.5 constituted three feature sets which were further applied to train the machine learning models. Correlation heat-maps for different feature sets were displayed separately in panel a (108 features), panel b (features with PPC < 0.8), and panel c (features with PPC < 0.5). Model performance was then evaluated in terms of ROC curves and PR curves to examine the feature redundancy (b and c).

sFig.4

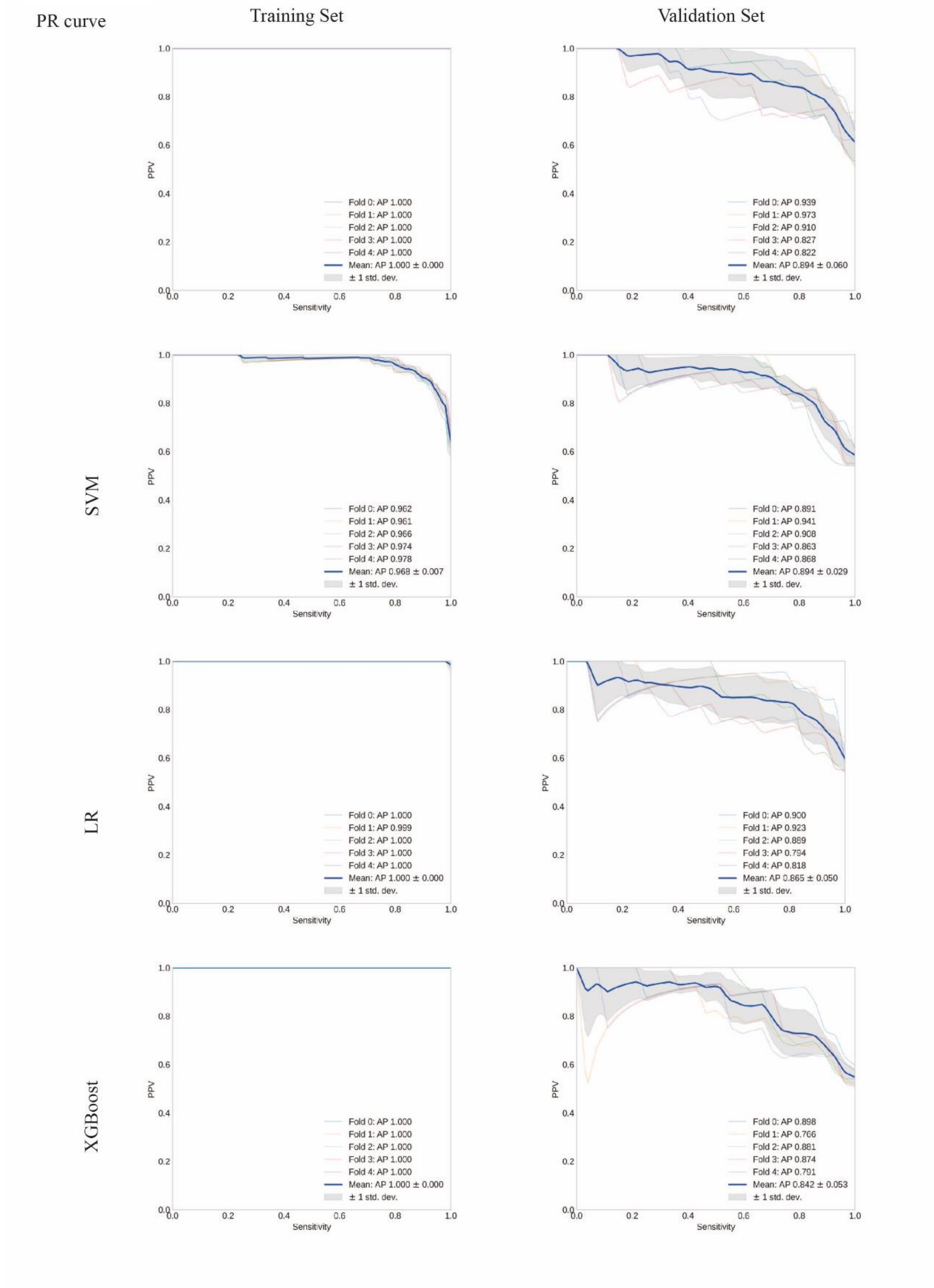

sFig.4. PR curve analyses of ML models on training and validation sets. To evaluate the performance of these ML models, PR curves were also analyzed along with ROC curves on

training and validation sets. Similarly, all ML models but SVM achieved PR-AUC of 1.000 on training set while MLP and SVM displayed a better performance on the validation set.

sFig.5

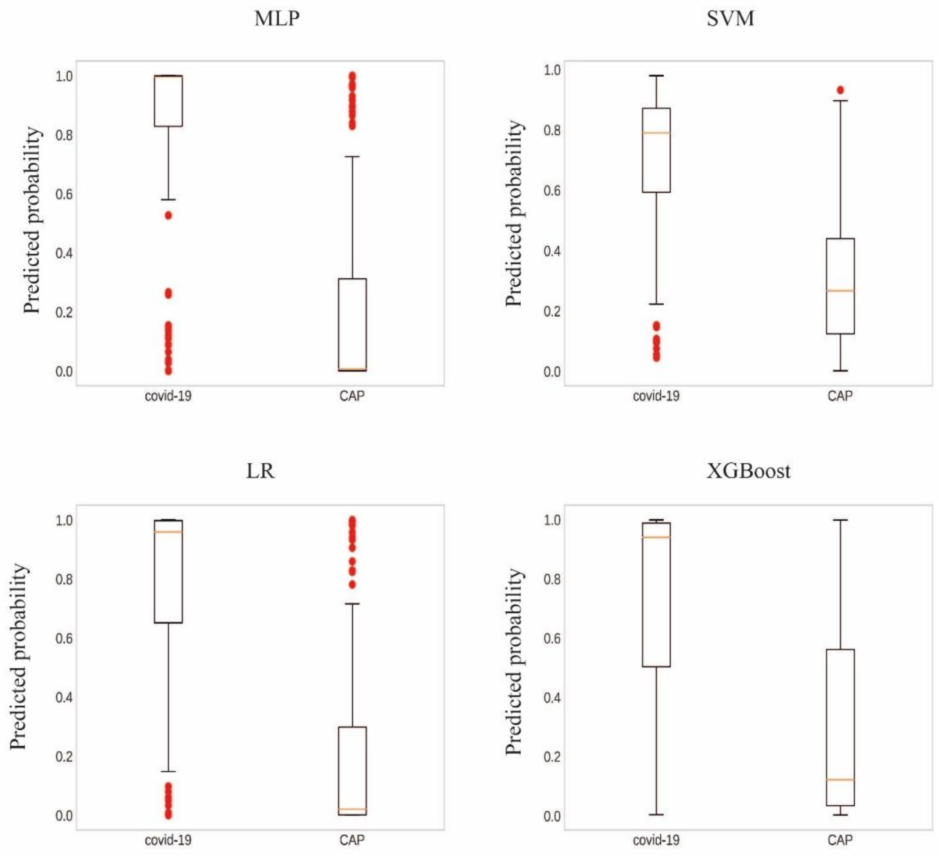

sFig.5. Predictive probabilities of COVID-19 by ML models. Predicted probabilities for COVID-19 and CAP (non-COVID-19) were quite differently distributed. A diagnostic threshold of 0.5 could well discriminate between these two classes.
