# Supplementary material for: A deep learning integrated radiomics model for identification of coronavirus disease 2019 using computed tomography
Source: Sci Rep. 2021 Feb 16;11:3938. doi: 10.1038/s41598-021-83237-6 (PMC7886892; doi:10.1038/s41598-021-83237-6)
Supplement: Supplementary file 2 — Supplementary Information 2. [file 41598_2021_83237_MOESM2_ESM.pdf]

Appendix Tables

Title: A Deep Learning Integrated Radiomics Model for Identification of Coronavirus Disease 2019 Using Computed Tomography

Authors: Xiaoguo Zhang[1§], Dawei Wang[2§], Jiang Shao[3], Song Tian[2], Weixiong Tan[2], Yan Ma[1], Qingnan Xu[1], Xiaoman Ma[1], Dasheng Li[4], Jun Chai[5], Dingjun Wang[6], Wenwen Liu[3], Lingbo Lin[3], Jiangfen Wu[2], Chen Xia[2], Zhongfa Zhang[1*]

Manuscript tracking number: SREP-20-02072A

Appended Table 1. Clinical stages of included COVID-19 patients

Type	Hospitals	Mild	Moderate	Severe	Critical
COVID-19	Medical Center 1 (60 cases)	18（30.0%）	41（68.3%）	1（1.7%）	0
	Medical Center 2（38 cases）	12 (31.6%）	25（65.8%）	1（2.6%）	0
	Medical Center 3（31 cases）	5（16.1%）	24（77.4%）	1（3.2%）	1（3.2%）

Appended Table 2. Imaging manifestations of included COVID-19 patients

Type	Hospitals	Multifocal small patchy shadows #	Ground glass opacity #	Consolidation #	Infiltrates #	Pleural effusion #	Interstitial changes #
COVID-19	Medical Center 1 (132 scans)	48（36.4%）	95（72.0%）	9（6.8%）	1（0.8%）	0	0
	Medical Center 2（38 scans）	8（21.1%）	28（73.7%）	0	0	0	2（5.3%）
	Medical Center 3（33 scans）	10（30.3%）	26（78.8%）	1（3.0%）	0	0	3（9.1%）
CAP	Medical Center 1 (96 scans)	35（36.5%）	16（16.7%）	2（2.1%）	0	0	49（51.0%）
	Medical Center 2（105 scans）	46（43.8%）	37（35.2%）	24（22.9%）	2（1.9%）	1（1.0%）	1（1.0%）
	Medical Center 3（62 scans）	32（51.6%）	38（61.3%）	23（37.1%）	0	9（14.5%）	3（4.8%）

Appendix Table 3. The number of radiomics features extracted with Pyradiomics2.2.0 on CT images.

Filters	Category							Total
	FOS	GLCM	GLRLM	GLSZM	NGTDM	GLDM	Shape#	
**Original image**	18	21	16	16	5	14	14	104
**Wavelet***	144	168	128	128	40	112	0	720
**Square**	18	21	16	16	5	14	0	90
**Square Root**	18	21	16	16	5	14	0	90
**Logarithm**	18	21	16	16	5	14	0	90
**Exponential**	18	21	16	16	5	14	0	90
**LocalBinaryPattern3D***	54	63	48	48	15	42	0	270
**Total**	288	336	256	256	80	224	14	1454

Note: #Category “Shape” features were only extracted from the original image, were not included in the group analysis because of the obvious difference between the study group and the control group;

*Filter “Wavelet” uses 8 levels of LLL, LLH, LHL, LHH, HLL, HHL, HLH, and HHH to process images;

*Filter “LocalBinaryPattern3D” uses 3 levels of m1, m2, and k to process images.

Appendix Table 4. Selected features with PCC<0.8

Number	Features
1	shape_LeastAxisLength_original
2	shape_MajorAxisLength_original
3	shape_Maximum2DDiameterColumn_original
4	shape_Maximum2DDiameterSlice_original
5	shape_Sphericity_original
6	firstorder_10Percentile_original
7	firstorder_Minimum_original
8	glcm_Autocorrelation_original
9	glcm_Imc2_original
10	gldm_LargeDependenceHighGrayLevelEmphasis_original
11	gldm_LowGrayLevelEmphasis_original
12	gldm_SmallDependenceHighGrayLevelEmphasis_original
13	glszm_SizeZoneNonUniformityNormalized_original
14	firstorder_Range_log-sigma-1-0-mm-3D
15	glcm_Correlation_log-sigma-1-0-mm-3D
16	glcm_Imc2_log-sigma-1-0-mm-3D
17	glrlm_LongRunEmphasis_log-sigma-1-0-mm-3D
18	firstorder_10Percentile_log-sigma-2-0-mm-3D
19	firstorder_InterquartileRange_log-sigma-2-0-mm-3D
20	firstorder_Median_log-sigma-2-0-mm-3D
21	firstorder_Skewness_log-sigma-2-0-mm-3D
22	glcm_ClusterShade_log-sigma-2-0-mm-3D
23	glcm_DifferenceVariance_log-sigma-2-0-mm-3D
24	glcm_Imc2_log-sigma-2-0-mm-3D
25	glrlm_RunEntropy_log-sigma-2-0-mm-3D
26	glszm_SizeZoneNonUniformityNormalized_log-sigma-2-0-mm-3D
27	firstorder_Skewness_log-sigma-3-0-mm-3D
28	glcm_Autocorrelation_log-sigma-3-0-mm-3D
29	glcm_ClusterProminence_log-sigma-3-0-mm-3D
30	glcm_Imc1_log-sigma-3-0-mm-3D
31	glszm_GrayLevelVariance_log-sigma-3-0-mm-3D
32	glszm_LargeAreaEmphasis_log-sigma-3-0-mm-3D
33	glszm_SmallAreaEmphasis_log-sigma-3-0-mm-3D
34	ngtdm_Strength_log-sigma-3-0-mm-3D
35	firstorder_Range_wavelet-LLH
36	firstorder_Skewness_wavelet-LLH
37	glcm_ClusterShade_wavelet-LLH
38	glcm_Correlation_wavelet-LLH
39	glcm_DifferenceVariance_wavelet-LLH
40	glcm_Idn_wavelet-LLH
41	gldm_LargeDependenceHighGrayLevelEmphasis_wavelet-LLH

42	firstorder_Maximum_wavelet-LHL
43	firstorder_Median_wavelet-LHL
44	glcm_ClusterShade_wavelet-LHL
45	glcm_Imc2_wavelet-LHL
46	firstorder_10Percentile_wavelet-HLL
47	firstorder_Maximum_wavelet-HLL
48	glcm_Correlation_wavelet-HLL
49	glcm_MaximumProbability_wavelet-HLL
50	gldm_LargeDependenceHighGrayLevelEmphasis_wavelet-HLL
51	glszm_GrayLevelVariance_wavelet-HLL
52	firstorder_Median_wavelet-HLH
53	glcm_Correlation_wavelet-HLH
54	glcm_Idm_wavelet-HLH
55	firstorder_Maximum_wavelet-HHL
56	glszm_SmallAreaLowGrayLevelEmphasis_wavelet-HHL
57	firstorder_Skewness_wavelet-HHH
58	gldm_DependenceVariance_wavelet-HHH
59	glszm_SizeZoneNonUniformityNormalized_wavelet-HHH
60	glszm_ZoneEntropy_wavelet-HHH
61	ngtdm_Contrast_wavelet-HHH
62	firstorder_Minimum_wavelet-LLL
63	gldm_LargeDependenceLowGrayLevelEmphasis_wavelet-LLL
64	firstorder_10Percentile_square
65	firstorder_TotalEnergy_square
66	glcm_InverseVariance_square
67	firstorder_Kurtosis_squareroot
68	ngtdm_Contrast_squareroot
69	glcm_Correlation_logarithm
70	glcm_Idmn_logarithm
71	glcm_InverseVariance_logarithm
72	gldm_DependenceVariance_logarithm
73	glrlm_LowGrayLevelRunEmphasis_logarithm
74	glcm_Correlation_exponential
75	glcm_Imc1_exponential
76	gldm_DependenceNonUniformityNormalized_exponential
77	gldm_DependenceVariance_exponential
78	glszm_GrayLevelNonUniformity_exponential
79	glszm_SizeZoneNonUniformityNormalized_exponential
80	glszm_SmallAreaEmphasis_exponential
81	ngtdm_Strength_exponential

PCC: Pearson Correlation Coefficient

Appendix Table 5. Selected features with PCC<0.5

Number	Features
1	shape_MajorAxisLength_original
2	shape_Sphericity_original
3	firstorder_10Percentile_original
4	firstorder_Minimum_original
5	glcm_Imc2_original
6	glcm_Correlation_log-sigma-1-0-mm-3D
7	glrlm_LongRunEmphasis_log-sigma-1-0-mm-3D
8	firstorder_Skewness_log-sigma-2-0-mm-3D
9	glcm_Imc2_log-sigma-2-0-mm-3D
10	glszm_LargeAreaEmphasis_log-sigma-3-0-mm-3D
11	firstorder_Skewness_wavelet-LLH
12	glcm_ClusterShade_wavelet-LLH
13	glcm_Correlation_wavelet-LLH
14	glcm_DifferenceVariance_wavelet-LLH
15	firstorder_Maximum_wavelet-LHL
16	glcm_ClusterShade_wavelet-LHL
17	firstorder_Maximum_wavelet-HLL
18	glcm_Correlation_wavelet-HLL
19	glcm_MaximumProbability_wavelet-HLL
20	gldm_LargeDependenceHighGrayLevelEmphasis_wavelet-HLL
21	glcm_Correlation_wavelet-HLH
22	firstorder_Skewness_wavelet-HHH
23	glszm_ZoneEntropy_wavelet-HHH
24	gldm_LargeDependenceLowGrayLevelEmphasis_wavelet-LLL
25	glcm_Correlation_exponential
26	gldm_DependenceVariance_exponential
27	glszm_SmallAreaEmphasis_exponential
28	ngtdm_Strength_exponential

PCC: Pearson Correlation Coefficient

Appendix Table 6. Full list of 108 selected features and its coefficient

Number	Features	Coefficient
1	shape_LeastAxisLength_original	1.880505066
2	shape_MajorAxisLength_original	2.294957641
3	shape_Maximum2DDiameterColumn_original	0.425688583
4	shape_Maximum2DDiameterSlice_original	1.627152883
5	shape_Sphericity_original	0.811862796
6	firstorder_10Percentile_original	0.793800597
7	firstorder_Minimum_original	0.184805094
8	glcm_Autocorrelation_original	-2.413475141
9	glcm_Imc2_original	1.03867076
10	gldm_LargeDependenceHighGrayLevelEmphasis_original	-3.031591956
11	gldm_LowGrayLevelEmphasis_original	-1.796036343
12	gldm_SmallDependenceHighGrayLevelEmphasis_original	-4.410486915
13	glrlm_LongRunHighGrayLevelEmphasis_original	-0.347583464
14	glszm_SizeZoneNonUniformityNormalized_original	-0.871195603
15	glszm_SmallAreaEmphasis_original	-0.127036391
16	firstorder_Range_log-sigma-1-0-mm-3D	-2.117761232
17	glcm_Correlation_log-sigma-1-0-mm-3D	2.26740875
18	glcm_Imc2_log-sigma-1-0-mm-3D	5.042339604
19	glrlm_LongRunEmphasis_log-sigma-1-0-mm-3D	1.266361312
20	firstorder_10Percentile_log-sigma-2-0-mm-3D	-0.626663897
21	firstorder_InterquartileRange_log-sigma-2-0-mm-3D	-1.140785943
22	firstorder_Median_log-sigma-2-0-mm-3D	-2.483727561
23	firstorder_Skewness_log-sigma-2-0-mm-3D	3.177975258
24	glcm_ClusterShade_log-sigma-2-0-mm-3D	0.237778839
25	glcm_DifferenceVariance_log-sigma-2-0-mm-3D	1.27653444
26	glcm_Imc2_log-sigma-2-0-mm-3D	-1.875571042
27	gldm_DependenceVariance_log-sigma-2-0-mm-3D	0.221343177
28	glrlm_RunEntropy_log-sigma-2-0-mm-3D	2.589981146
29	glrlm_RunVariance_log-sigma-2-0-mm-3D	2.478212614
30	glszm_SizeZoneNonUniformityNormalized_log-sigma-2-0-mm-3D	1.888927292
31	firstorder_10Percentile_log-sigma-3-0-mm-3D	-0.885390748
32	firstorder_Skewness_log-sigma-3-0-mm-3D	3.297306764
33	glcm_Autocorrelation_log-sigma-3-0-mm-3D	-1.165320669
34	glcm_ClusterProminence_log-sigma-3-0-mm-3D	-1.320963385
35	glcm_DifferenceVariance_log-sigma-3-0-mm-3D	2.14962571
36	glcm_Imc1_log-sigma-3-0-mm-3D	0.16793972
37	glrlm_RunEntropy_log-sigma-3-0-mm-3D	1.21665584
38	glszm_GrayLevelVariance_log-sigma-3-0-mm-3D	0.169704864
39	glszm_LargeAreaEmphasis_log-sigma-3-0-mm-3D	1.086798968
40	glszm_LargeAreaLowGrayLevelEmphasis_log-sigma-3-0-mm-3D	1.047031209
41	glszm_SmallAreaEmphasis_log-sigma-3-0-mm-3D	0.525500887

42	glszm_ZoneVariance_log-sigma-3-0-mm-3D	2.687815373
43	ngtdm_Busyness_log-sigma-3-0-mm-3D	2.416717522
44	ngtdm_Strength_log-sigma-3-0-mm-3D	1.457640141
45	firstorder_Range_wavelet-LLH	-1.361441577
46	firstorder_Skewness_wavelet-LLH	0.149274045
47	glcm_ClusterShade_wavelet-LLH	1.086698727
48	glcm_Correlation_wavelet-LLH	-0.712348875
49	glcm_DifferenceVariance_wavelet-LLH	0.381594775
50	glcm_Idn_wavelet-LLH	-0.135303041
51	gldm_LargeDependenceHighGrayLevelEmphasis_wavelet-LLH	-1.778343411
52	glszm_SizeZoneNonUniformityNormalized_wavelet-LLH	-2.033921119
53	firstorder_Maximum_wavelet-LHL	-0.073670032
54	firstorder_Median_wavelet-LHL	0.913598927
55	glcm_ClusterShade_wavelet-LHL	-0.964534434
56	glcm_Imc2_wavelet-LHL	-1.080561542
57	ngtdm_Busyness_wavelet-LHL	-0.32845217
58	firstorder_10Percentile_wavelet-HLL	-1.31190271
59	firstorder_Maximum_wavelet-HLL	-0.158519493
60	glcm_Correlation_wavelet-HLL	-2.359568048
61	glcm_Imc2_wavelet-HLL	-4.491543677
62	glcm_MaximumProbability_wavelet-HLL	0.050356873
63	gldm_LargeDependenceHighGrayLevelEmphasis_wavelet-HLL	0.424853177
64	glszm_GrayLevelVariance_wavelet-HLL	3.440753477
65	firstorder_Median_wavelet-HLH	-0.704105121
66	glcm_Correlation_wavelet-HLH	-0.006522434
67	glcm_Imc2_wavelet-HLH	0.022906707
68	glcm_Idm_wavelet-HLH	-0.706327233
69	glcm_InverseVariance_wavelet-HLH	-1.133511664
70	firstorder_Maximum_wavelet-HHL	3.296839961
71	gldm_DependenceVariance_wavelet-HHL	0.26944139
72	glrlm_RunLengthNonUniformity_wavelet-HHL	0.799816899
73	glszm_SmallAreaLowGrayLevelEmphasis_wavelet-HHL	1.974890702
74	firstorder_Skewness_wavelet-HHH	-0.340716895
75	gldm_DependenceVariance_wavelet-HHH	-1.006619688
76	glszm_SizeZoneNonUniformityNormalized_wavelet-HHH	0.486293033
77	glszm_ZoneEntropy_wavelet-HHH	-0.06150977
78	ngtdm_Contrast_wavelet-HHH	-0.480069748
79	firstorder_Minimum_wavelet-LLL	0.033769647
80	gldm_LargeDependenceLowGrayLevelEmphasis_wavelet-LLL	-0.205225284
81	glszm_SizeZoneNonUniformityNormalized_wavelet-LLL	-1.10151859
82	firstorder_10Percentile_square	-0.782584428
83	firstorder_TotalEnergy_square	-2.299864375
84	glcm_InverseVariance_square	0.788783461
85	firstorder_Kurtosis_squareroot	-1.083923784

86	firstorder_Skewness_squareroot	-0.548422833
87	ngtdm_Contrast_squareroot	-0.079778824
88	firstorder_Kurtosis_logarithm	-0.757394057
89	glcm_Correlation_logarithm	-1.064693375
90	glcm_Idmn_logarithm	0.525876702
91	glcm_InverseVariance_logarithm	1.278209203
92	gldm_DependenceVariance_logarithm	-0.604391038
93	glrlm_LowGrayLevelRunEmphasis_logarithm	-3.781826392
94	glcm_Correlation_exponential	-0.794084783
95	glcm_Imc1_exponential	-0.167110841
96	gldm_DependenceNonUniformityNormalized_exponential	0.038363741
97	gldm_DependenceVariance_exponential	-2.254036224
98	glrlm_LongRunEmphasis_exponential	-3.474263908
99	glrlm_LongRunHighGrayLevelEmphasis_exponential	-3.274788077
100	glrlm_LongRunLowGrayLevelEmphasis_exponential	-3.156878333
101	glrlm_ShortRunEmphasis_exponential	-0.899122026
102	glrlm_ShortRunLowGrayLevelEmphasis_exponential	-1.019911454
103	glszm_GrayLevelNonUniformity_exponential	-2.741863539
104	glszm_SizeZoneNonUniformityNormalized_exponential	2.67762321
105	glszm_SmallAreaEmphasis_exponential	-0.32787846
106	glszm_SmallAreaLowGrayLevelEmphasis_exponential	-0.676263749
107	glszm_ZoneVariance_exponential	2.638052411
108	ngtdm_Strength_exponential	-1.218921942
